# Study on the Evolution Mechanism of Ecosystem Services in Karst Mountainous Areas from the Perspective of Humanities

**DOI:** 10.3390/ijerph192013628

**Published:** 2022-10-20

**Authors:** Peipei Miao, Xiaoqing Zhao, Junwei Pu, Pei Huang, Xiaoqian Shi, Zexian Gu

**Affiliations:** 1 School of Earth Sciences, Yunnan University, Kunming 650500, China; 2 Institute of International Rivers & Eco-Security, Yunnan University, Kunming 650500, China; 3 Forest Resource Management Division, Nujiang Forestry and Grassland Administration, Lushui 673100, China

**Keywords:** climate change, impact of implementation of ecological engineering, farmers’ behavior and willingness, social and economic activities, multiple logistic model, Yunnan karst mountain area

## Abstract

Anthropogenic activities have altered ecosystem service functions in the karst mountainous areas. The implementation of ecological restoration projects by the government, the behavior, attitude, and willingness of farmers to participate in their implementation, the application of pesticides and fertilizers, in addition to other socio-economic activities, have had a significant impact on the ecosystem services (ESS) of the region. Taking Guangnan County, a typical karst mountainous area in Yunnan Province, as an example, this study analyzes the evolutionary characteristics of six types of ESS and the driving mechanism of the change in ESS from the anthropogenic macro and micro perspective using questionnaire surveys and the multivariate logistic model. The results showed that (1) ecological restoration projects in the past 20 years have promoted an overall ecological transformation in the typical karst mountainous areas of the Yunnan Province (2) from the macro perspective, and the implementation of such ecological projects is beneficial in increasing soil conservation, carbon sequestration, habitat support, and cultural services. The reduction in agricultural population is beneficial in improving habitat support services, and the increase in the annual average tourism income and the tertiary industry is beneficial in increasing cultural services. Among them, the impact of hydraulic engineering on water production and the tertiary industry on cultural services are the most significant, with the change in the human disturbance index having the most substantial impact on soil conservation, carbon sequestration, and habitat support (3) at the micro level. Increasing pesticide and fertilizer application, willingness and use by farmers has a positive impact on food supply and a negative impact on habitat quality. An increase in the number and willingness of farmers participating in restoring farmland to forests and water conservancy projects was observed. This has a positive impact on soil conservation, water production, and carbon sequestration. Among them, the application of chemical fertilizers and pesticides has the most significant impact on food supply and habitat support, and the willingness to implement the projects on restoring farmlands to forests has the most significant impact on carbon sequestration. The willingness to implement terracing has the greatest impact on water production and soil conservation, and aesthetic value has the greatest impact on cultural services.

## 1. Introduction

Ecosystems provide a variety of services for human survival, health, and well-being [1,2,3]. Human survival and development are inseparable from the multiple services provided by ecosystems [4]. However, the increasingly negative impact of human activities on the ecosystem has resulted in severe damage to ESS [5,6]. It is thus essential to explore the driving mechanism of ESS change due to anthropogenic activities [7,8]. In this regard, significant research has been carried out on the classification, evaluation, and driving mechanisms of ESS [9,10], which has achieved remarkable results. For example, launched in 2019, Intergovernmental Science-Policy Platform on Biodiversity and Ecosystem Services (IPBES) carried out a study on the drivers of ESS change, which divided the factors affecting ESS into direct and indirect drivers [11]. This will aid individuals in understanding their unsustainable behavior and provide a scientific basis for the formulation of ESS function recovery measures [12,13,14].

Scholars typically select macro factors such as land-use change, policy, and economy while studying the driving mechanisms of climate change [15,16,17]. However, micro-level driving factors are seldom considered, mainly considering factors such as household ecological engineering, compensation income, and the proportion of household fossil fuel use [18,19]. There are even fewer studies considering the impact of farmers’ willingness, behavior, and attitude on the changes in ESS [20,21,22]. Although the implementation of government policies such as ecological restoration projects is beneficial in increasing ESS to a certain extent, the behavior and willingness of farmers to participate in these projects will directly affect implementation, thus affecting the change in ESS.

The study of the driving mechanisms of climate change should therefore combine macro as well as micro factors and strengthen the comprehensiveness and integrity of the selection of the anthropogenic driving factors. At present, the anthropogenic driving factors are studied either via qualitative or quantitative analysis, with the combination of the two research methods rarely being used [23,24,25]. In particular, quantitative methods such as grey correlation analysis and correlation analysis require a linear relationship between the independent and dependent variables. Affected by multicollinearity, autocorrelation, and heteroscedasticity, linear regression is sensitive to outliers, which will severely affect the regression line and, ultimately, the predicted value. The multivariate logistic model [26], on the other hand, does not require a linear relationship between the dependent and independent variables. The model can divide the dependent and independent variables into continuous and categorical variables. The multivariate logistic model was therefore used in this study to analyze the driving factors of ESS change.

The unsustainable behavior of human beings in the karst mountainous areas has damaged ecosystem functions and severely threatened social production and development over several years [27,28,29,30,31]. The government has thus implemented a series of ecological restoration projects, such as projects on terracing, water conservation, and restoring farmlands to forests. However, the willingness of the farmers to implement the project and the differences in production methods also affect the effectiveness of the project implementation, thus changing the ESS. Therefore, Guangnan County, a mountainous area in Yunnan Province with typical karst human-land system characteristics, was selected as the study area to analyze the spatial and temporal variation characteristics of ESS to qualitatively analyze the influencing factors of ESS variation from macro to micro levels. Based on the multivariate logistic model, this study quantitatively reveals the significant anthropogenic factors that cause the change in ESS and provides the basis for the formulation of ecological restoration measures in the ecologically fragile areas of the karst region.

## 2. Materials and Methods

### 2.1. Study Area

**Figure 1:**
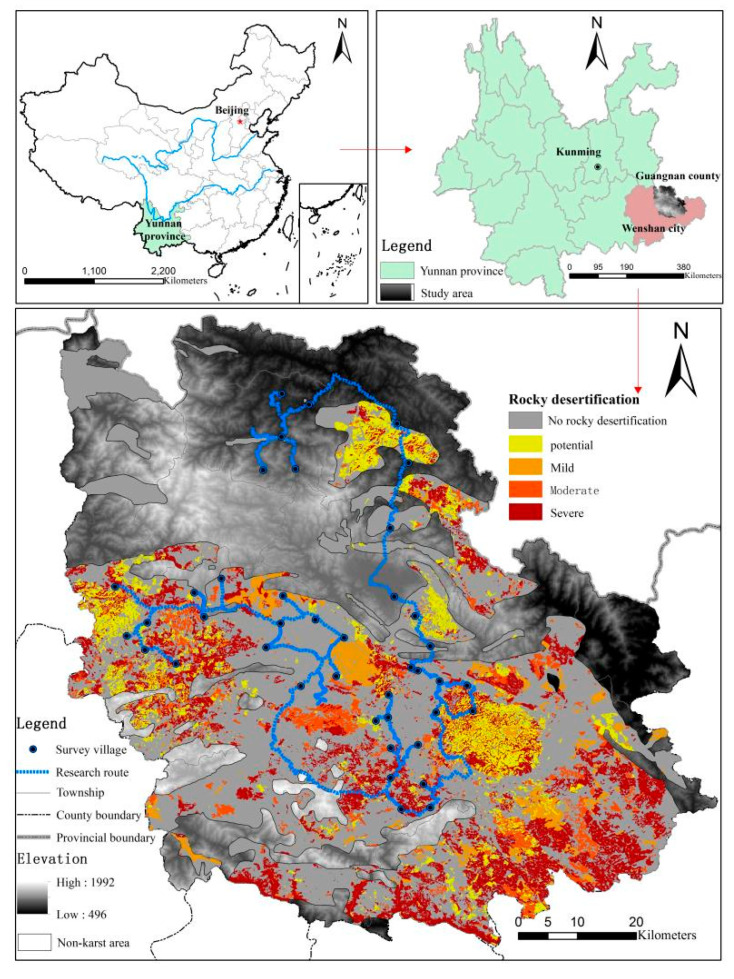
Study area.

Guangnan County is located in the southeast region of the Yunnan Province (104°31′~105°39′ E, 23°29′~24°28′ N), which belongs to the middle and southern subtropical plateau monsoon climate. The annual average sunshine is 1857.7 h, the annual average temperature is 16.7 °C, and the annual average rainfall is 1056.5 mm. The dry and rainy seasons are obvious. Karst landforms are widely distributed, accounting for 74% of the total area of the county, mainly located in the western, southern, and south-central regions. The rocky desertification area covers 1840.53 km^2^ (23.81%) of the total area of the county, which is one of the 200 major rocky desertification counties in China. The soil types are mainly composed of red soil and limestone soil, and the vegetation is primarily composed of Yunnan pine, oak, and Chinese fir, with rich species. The water resources in the area are fairly abundant, with a relatively low utilization rate. There are 11 ethnic minorities in the county, accounting for 61.8%. In 2018, the total population of the county was 822,600, of which the agricultural population accounted for 91.29%. In 2018, the proportion of GDP of the primary, secondary, and tertiary industries in Guangnan County was 30.22:30.34:39.44, respectively [32] (Figure 1).

### 2.2. Research Methods

According to the natural and cultural conditions in the study area and the results of the literature review, six types of ESS were selected for accounting.

#### 2.2.1. ESS Accounting Methods

(1) Accounting of food supply

The food supply service was spatially expressed as per the NDVI. The formula used is as follows [33,34,35,36]:

(1) $$G_{i}=\frac{NDVI_{i}}{NDVI_{sum}}\times G_{SUM}$$

In the formula, $G_{i}$ is the grain output of the *i*th grid; $G_{SUM}$ is the total grain output; $NDVI_{i}$ is the NDVI value of the *i*th grid; $NDVI_{SUM}$ is the total NDVI value of cultivated land and grassland.

(2) Accounting of water yield

The water production was estimated using the InVEST model [37,38]. The formula is presented as follows:

(2) $$Y\left(x\right)=\left(1-\frac{AET\left(x\right)}{P\left(x\right)}\right)\times P\left(x\right)$$

In the formula, *Y* (*x*) is the water yield (mm) on the pixel X, *AET* (*x*) is the actual evapotranspiration on image X (mm), and *P* (*x*) is the average annual rainfall on image X (mm).

(3) Accounting of soil conservation

The soil loss equation (RUSLE) model was used for estimating soil conservation [39,40,41,42].

(4) Accounting of carbon sequestration

The CASA model was used to calculate the Net Primary Productivity (NPP) through which the amount of carbon sequestration was calculated. The formula used is as follows [43,44,45].

(3) NPP(x, t)=APAR(x, t) × ε(x, t)

(4) $$V_{c}=1.63\times P_{c}\times \overset{n}{\underset{i=1}{\sum }}NPP_{i}S_{i}$$

In the formula, APAR(x, t) is the photosynthetically active radiation absorbed by pixel X in month t (MJ/m^2^); ε(x,t) is the actual light energy utilization rate of pixel in month t (gC/m^2^). $V_{c}$ is the value of carbon sequestration, and $P_{c}$ is the price of fixed carbon dioxide.

(5) Accounting of habitat support

Habitat support services were evaluated based on the InVEST model, which is formulated as follows [46,47,48,49]:

(5) $$Q_{xj}=H_{j}\left[1-\left(\frac{D_{xj}^{z}}{D_{xj}^{z}+K^{2}}\right)\right]$$

where *Q_xj_* is the habitat quality of land use type *j* at grid unit *x*, *H_j_* is the score of the corresponding habitat type of land use type *j*, *D_xj_* is the habitat stress level, and *K* is a half-saturation constant.

(6) Accounting of cultural value

The tourism value was selected as the evaluation object of ecosystem cultural services based on the direct use value method [50]. The formula is presented as follows:

(6) $$V_{t}=V_{t}\left(a\right)+V_{t}\left(b\right)\;V_{t}\left(a\right)=\sum^{\;}A_{i}\times P_{i}\;V_{t}\left(b\right)=\sum^{\;}B_{i}\times P_{i}$$

where $V_{t}$ is the total value of cultural services, $V_{t}\left(a\right)$ is the accessibility value of scenic spots, $V_{t}\left(b\right)$ is the visibility value of scenic spots, $A_{i}$ represents different pixels grouped by the number of visible scenic spots according to visibility, $P_{i}$ is the annual tourism revenue (CNY/ha), and $B_{i}\;$ are different pixels grouped by the distance from scenic spots according to accessibility.

#### 2.2.2. Questionnaire Survey Method

The questionnaires were designed to assess the behavior, attitude, and willingness of the farmers to implement ecological restoration projects, to assess production and life, and to collect related data for the analysis of micro-driving factors of ESS change. The questionnaires were tested, revised, and improved, before 514 questionnaires were completed in August and September 2019. Out of these, 468 (91.05%) valid, complete questionnaires were obtained [51,52,53,54].

#### 2.2.3. Multivariate Logistic Regression Model

Suitable index factors were selected as independent variables to analyze the changes in each ESS. The changes in each ESS were considered as dependent variables. The multiple logistic regression model [26] was then used to determine the most significant driving factors that affect the changes in each ESS.

### 2.3. Data Source and Processing

Refer to Table 1 for the data sources and data processing required for the analysis of the social and anthropogenic driving forces in Guangnan County.

## 3. Analysis of Results

### 3.1. Spatial and Temporal Distribution Pattern of ESS

The average annual food supply and cultural value increased yearly from 2000 to 2018, with an average annual increase of 1.59 t/ha and 4.61 CNY/ha, respectively. The annual average water yield, soil conservation, and carbon sequestration showed an initial decreasing and then increasing trend, with an overall annual average increase of 17.03 mm and 129.67 t/ha, respectively, and an annual average decrease of 84.33 CNY·ha^−1^·a^−1^. The annual average habitat quality, on the other hand, showed a yearly decreasing trend, with an annual average decrease of 0.07 (Table 2).

**Figure 2:**
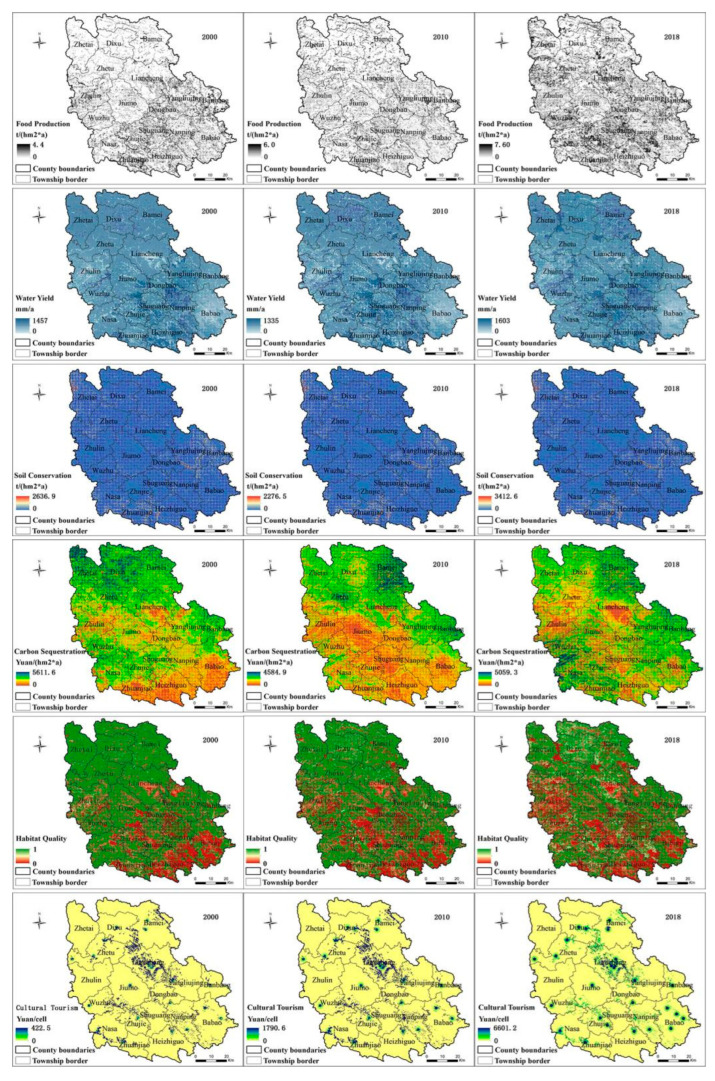
ESS Space Map.

The soil holding capacity increased more in the northwest and south and decreased in the southeast from 2000 to 2018. The value of carbon sequestration increased in the east and southwest regions and decreased more in the northwest, central and south-central regions. The habitat quality increased in the east and southeast and decreased in the west and northwest. The value of cultural services increased in the central region and decreased in the western and northwestern regions. Spatially, there was an increase in the food supply in the central, south-central, and southeastern regions and a decrease in the western and eastern regions. The water yield showed an increase in the north, central and south-central regions and a decrease in the northwest, south, and southeast regions (Figure 2).

### 3.2. Analysis of the Impact of Macro Factors Based on the Changes in ESS at the Socio-Economic Level

**Figure 3:**
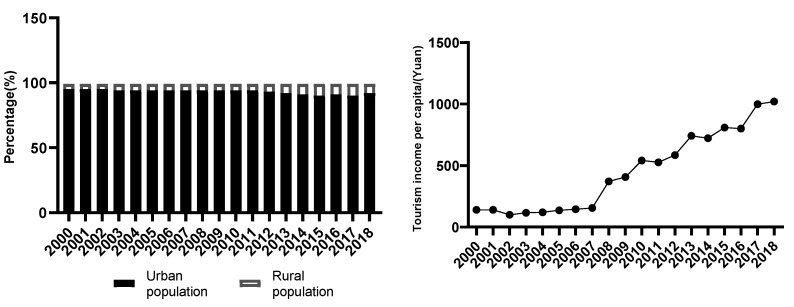
Change in urban population and agricultural population (**left**) and change in per capita tourism income (**right**).

The total population of Guangnan County showed an increasing trend from the years 2000 to 2018. However, the proportion of the agricultural population showed a significant downward trend. Among them, the proportion of the agricultural population decreased by 4.5% (Figure 3). This decrease is primarily due to the out-migration of the labor force, which led to several sloping farmlands becoming barren, the increase in the vegetation coverage of the original land, and a gradual improvement of the ecological environment. The decline in the proportion of the agricultural population is conducive to soil conservation and the increase in habitat support services.

The total number of tourists received by several scenic spots in Guangnan County saw an increasing trend from 2000 to 2018, demonstrating an increase by a total number of 2.84 million tourists and an average annual growth of 157,700 tourists (Figure 3). There was a consequent increase in the comprehensive income, with an average annual growth of CNY 159 million [19]. With the continuous development in the economy, the annual per capita tourism income in the study area increased from CNY 149 to CNY 978. This increase is beneficial to cultural value services.

**Figure 4:**
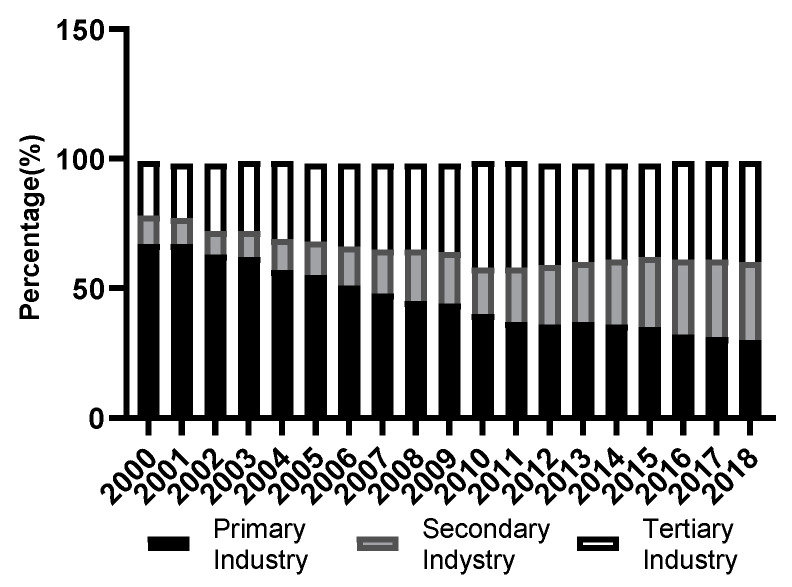
Change in industry proportion.

In 2000, the economy of Guangnan County was dominated by primary industry. The proportion of gross output value of primary, secondary, and tertiary industries is 67.14:10.94:21.92, respectively. Since 2000, the tertiary industry has rapidly developed under the promotion of the western development strategy. In 2018, the economy of Guangnan County showed a significant decline in the primary industry and an increase in the secondary and tertiary industries. The proportion of the gross output value of the primary, secondary, and tertiary industries was 30.22:30.34:39.44, respectively (Figure 4). With the continuous optimization of industrial structure, the proportion of agriculture is decreasing, the proportion of the light industry and especially that of the service industry is increasing, and the driving role of tourism in the tertiary industry is enhanced. The adjustment of industrial structure can reduce the consumption of energy and materials, play an important role in the protection of the ecological environment, and aid in increasing the habitat support and cultural value services in the study area.

### 3.3. Analysis of the Impact of Micro-Factors on ESS Change at the Household Level

#### 3.3.1. Influence of Farmers’ Behavior, Attitude, and Willingness to Implement the Project on ESS

(1) Farmers’ behavior, attitude and willingness to implement slope-to-ladder projects on ESS

**Figure 5:**
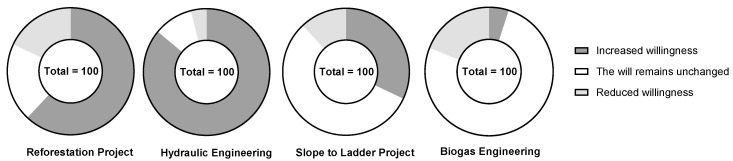
Farmers’ willingness to implement various projects.

**Figure 6:**
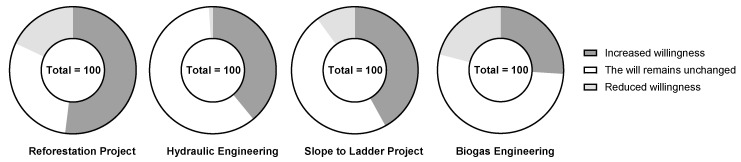
Farmers’ attitudes to the benefits of various projects.

After the implementation of the slope-to-ladder project, the number of farmers participating in the project showed a decreasing trend from 2000 to 2018, decreasing by 9 households and accounting for 2.2% of the total households (Table 3). If the government were to stop subsidizing the project, 56% of farmers’ willingness to implement the project would remain unchanged (Figure 5). According to the survey, the implementation of the peasant household project was mainly concentrated in the period 2000–2010, when a large number of young and middle-aged laborers were primarily engaged in farming at home. However, the number of peasant households participating in the project declined in the period 2010–2018. This was due to the lack of rural labor force due to out-migration of young and middle-aged laborers, coupled with the lack of management during the implementation of the slope to terrace project, which limited and affected the efficient implementation of the project. Therefore, the number of farmers participating in the slope-to-ladder project decreased, but their willingness remained unchanged. Of the farmers, 42% believed that the grain yield, water, and soil conservation effect of cultivated land increased significantly after the implementation of the slope-to-ladder project (Figure 6). Overall, the implementation of the slope-to-ladder project is found to be beneficial to soil conservation and the increase in food supply services in the study area.

(2) Farmers’ behaviors, attitudes and willingness to implement the fallow forestry project on ESS

Since the implementation of the fallow forestry project, there was an increase in the number of participating households from 2000 to 2018, with 140 (34.40%) new households participating (Table 3). If the government were to stop subsidizing the Fallow Forestry Project, 62% of the households would increase their willingness to implement the project (Figure 5). The main reason is that the phenomenon of deforestation and land reclamation was common knowledge before the implementation of the project. With the out-migration of the main labor force in the village, the surplus labor force of the elderly and children was limited, which led to several sloping farmlands being abandoned. More farmers voluntarily participated in the project, which gradually improved the ecological environment. Secondly, before the implementation of the project, farmers needed to cut a significant amount of firewood to cook food and pig fodder. The cooked food for livestock was gradually replaced with raw corn flour or green feed, reducing the use of firewood. At the same time, in the Zhuang villages, the houses were mainly made of wood in the past, where a large amount of wood was required to be cut down. Now, the building materials comprise mostly cement, greatly reducing the demand for wood. Therefore, the reduction in wood consumption by farmers protected the vegetation in the mountain, which is beneficial to the vegetation growth and to the implementation of the fallow forestry project.

With the increasing number of farmers participating in the projects, the forest land has been well protected, which is conducive to vegetation restoration. A total of 52% of the farmers believed that the vegetation coverage on the mountain increased, and the phenomenon of soil erosion significantly decreased after the implementation of the project (Figure 6). The behavior and attitude of the farmers toward the implementation of the project of restoring farmland to forests are therefore conducive to the increase in soil conservation, carbon sequestration, habitat, and cultural services of the ecosystem.

(3) Farmers’ behaviors, attitudes and willingness to influence ESS regarding hydraulic engineering implementation

Since the implementation of hydraulic engineering, the number of farmers participating in the project showed an increasing trend from 2000 to 2018, with 237 (58.23%) new participating households (Table 3). If the government were to stop subsidizing the project, the willingness of 86% of the farmers would increase to implement the project (Figure 5). As per the questionnaire survey, the majority of the new farmers are located in the southern part of the study area, which is a moderate and severe area of karst rocky desertification, where it is difficult to store water. Therefore, the number and the willingness of farmers to participate in water projects showed an increase. Of the farmers, 39% believed that it would be more convenient to produce, use and store water after the implementation of hydraulic engineering (Figure 6). Therefore, the hydraulic engineering is conducive to regulating the effective use of water resources.

(4) Farmers’ behaviors, attitudes and willingness to implement biogas projects on ESS

After the implementation of biogas projects, the number of farmers participating in the project showed a decreasing trend from 2000 to 2018, decreasing by 61 households and accounting for 13.03% (Table 3). If the government were to stop subsidizing the project, only 4% of the farmers’ willingness to implement the project were to remain unchanged (Figure 5). The implementation of biogas projects was mainly concentrated from 2006 to 2010. During this period, the government implemented biogas projects to change the way farmers use energy, from using firewood to biogas, primarily to reduce deforestation. From 2010 to 2018, the number of farmers participating in biogas projects declined, mainly due to the lack of rural labor force caused by the out-migration of the young and middle-aged labor force. With the gradual shrinking of the rural courtyard economy, several farmers abandoned the breeding industry, resulting in a shortage of raw materials for biogas. Additionally, with the transformation of the rural power grid, the cost of electricity in rural areas decreased, coupled with the popularization of electrical appliances, rendering some biogas digesters idle. Of the farmers, 26% believe that there is an increase in the vegetation cover on the mountain after the implementation of the biogas project (Figure 6). The implementation of the biogas project will therefore be beneficial in increasing the habitat support services in the study area.

#### 3.3.2. Effect of the Attitude and Willingness to Implement the Application of Pesticide and Fertilizer

(1) That effect of attitude and willingness to implement the application of pesticides

**Figure 7:**
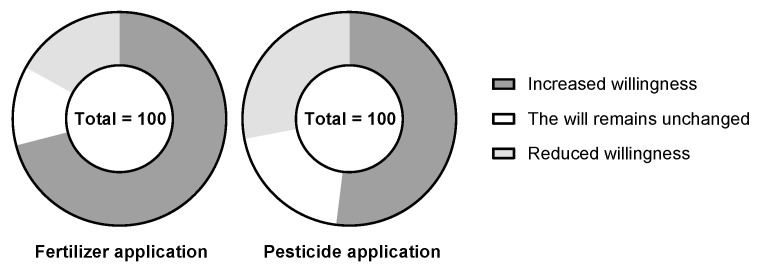
Farmers’ willingness to apply chemical fertilizers (**left**) and pesticides (**right**).

**Figure 8:**
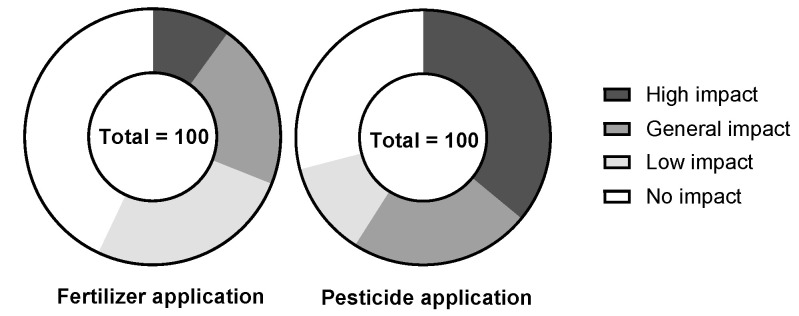
Farmers’ attitudes towards the impact of chemical fertilizer application (**left**) and pesticide application (**right**) on the ecological environment.

The average application of pesticides per mu of cultivated land showed a yearly increasing trend from 2000 to 2018 (Table 4). The number of new farmers using pesticides was 226, accounting for 55.52% (Table 4). If the price of pesticides were to be increased, it was found that only 28% of farmers would be willing to reduce the application amount (Figure 7). Due to the increasing number of crop diseases and insect pests, in addition to drug resistance, farmers can only rely on increasing the amount of pesticide application to reduce the harm crops and to increase production. By and large, most farmers were reluctant to reduce the number of pesticides used. However, with the yearly increase in pesticide application, 36% of farmers believed that the increasing pesticide application has an impact on the ecological environment (Figure 8). This shows that farmers have realized the negative impact of pesticides on the ecological environment while improving crop yields.

(2) The effect of attitude and willingness to implement the application of chemical fertilizers on ESS

The average amount of chemical fertilizers applied per mu of cultivated land showed a yearly increasing trend from 2000 to 2018 (Table 4). The number of new farmers using chemical fertilizer was found to be 223 (54.79%) (Table 4). If the price of chemical fertilizers were to increase, only 17% of farmers would be willing to reduce the application of chemical fertilizers (Figure 7). The survey found that the economic conditions in the study area were poor around the year 2000, with the farmers having a low income. However, since the number of livestock raised by farmers was large, the major source of fertilizer was livestock manure. There has been a decrease in the number of livestock fed by farmers over the past 10 years or so, due to the increased number of migrant workers, which has reduced the source of livestock manure. Therefore, farmers have had no choice but to increase the application of chemical fertilizers to increase crop production. However, even though the amount of chemical fertilizer increases yearly, 10% of the farmers believe that the amount of chemical fertilizer has a negative impact on the ecological environment, such as soil hardening (Figure 8).

#### 3.3.3. Influence of Planting Behavior, Attitude, and Willingness on ESS

**Figure 9:**
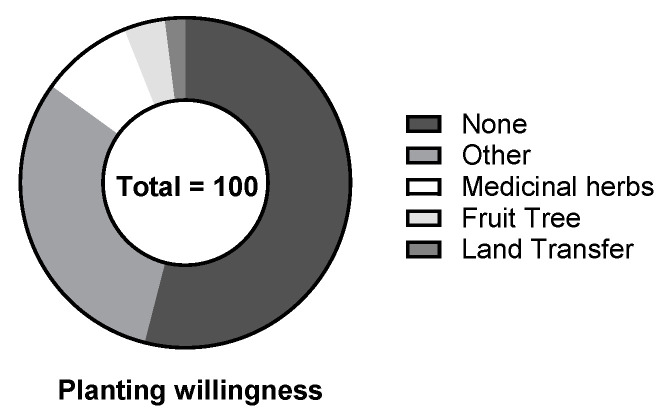
Farmers’ planting intention.

According to the survey, 54% of the farmers remained unchanged in their willingness to grow crops, and they continued to grow traditional food crops such as corn and rice from 2000 to 2018. This is primarily because the farmers were unable to replant suitable crops, due to technical or financial constraints. In addition, with the increase in the number of migrant workers, the remaining farmers were mostly elderly who were unable to replant, due to limitations in their energy and physical ability. They could, therefore, only grow traditional crops such as corn to be self-sufficient and to maintain their basic life. Of the farmers, 31% were found to prefer to grow other crops with higher economic benefits, followed by 9% of the farmers who were willing to grow medicinal plants, 4% of the farmers who were willing to grow fruit trees, and only 2% of farmers were willing to transfer their land if conditions permit (Figure 9). Supply services and habitat support services benefit to some extent, as farmers tend to diversify crop production.

### 3.4. Analysis of the ESS Evolution Mechanism Based on the Multivariate Logistic Model

#### 3.4.1. Selection of Human Driving Factors

According to the results of the qualitative analysis, statistical yearbooks, and related reports, the study focused on four factors—social, behavioral, economic, and cultural. The macro perspective was selected from social, behavioral, and economic factors. Among them, the social factors included change in population density (X1), change in agricultural population density (X2), change in the implementation of slope-to-ladder projects (X3), change in the implementation of hydraulic engineering (X4), change in the implementation of restoring farmlands to forests projects (X5), and change in the implementation of biogas projects (X6). The human disturbance index change (X7) was selected as the behavioral factor. The economic factor comprised the change in farmers’ net income (X16) and the change in the proportion of the total value of three industries (X17), (X18), and (X19). The micro-perspective was selected from the cultural and behavioral factors. Among them, the cultural factors included aesthetic value (X20), scientific research value (X21), tourism value (X22), and historical and cultural heritage value (X23). Behavioral factors included the change in the application amount of chemical fertilizers and pesticides (X8), the change in the application amount of chemical fertilizer (X9), the implementation intention (X10), the implementation intention of pesticides (X11), implementation intention of the biogas projects (X12), the willingness in the implementation of slope-to-ladder projects (X13), the willingness in the implementation of hydraulic engineering (X14), and the willingness in the implementation of restoring farmlands to forests projects (X15) (Table 5). Value is a categorical variable, where “0” is defined as a decrease in value and “1” as an increase in value.

Firstly, the index of a continuous variable is dimensionless. Using SPSS software for the collinearity test, it was found that the independent variable population density and agricultural population density had a collinearity test (VIF) value greater than 10. It was, therefore, necessary to eliminate the population density factor and select agricultural population density for the model calculation. The significant level (sig) in the likelihood ratio test was used to test the goodness of fit of the model. If sig was less than 0.05, the fitting effect of the model is considered to be good. The greater the Wald value or smaller the significance level (sig), the more significant the independent variable.

#### 3.4.2. Results of the Multiple Logistic Regression Model

In the definition of the change in the dependent variable ESS, “1” signified a decrease in the ESS, “2” signified no change, and “3” signified an increase in the ESS. In the case of food supply services, soil conservation services, water production services, carbon sequestration services, and habitat support services as dependent variables, 19 driving factors were selected, while 22 driving factors were selected when the dependent variable was cultural services.

The dependent variables in the model results represented the reduction in food supply services, the reduction in water production services, and the decrease in soil from 2000 to 2018. When soil conservation services, carbon sequestration services, habitat support services, and cultural services decreased, the significant level of the likelihood ratio test in the simulation fitting information was 0.000, which is less than 0.05, indicating that the model fits well (Table 6).

In the analysis results of food supply services from 2000 to 2018, the order of influence on food supply services was as follows: change in chemical fertilizers application amount > change in pesticide application amount > the implementation intention of chemical fertilizers > change in the implementation of slope-to-ladder project > proportion change in primary industry > the implementation intention of pesticides. Among them, the Wald value (21.051) of the change in the application rate of chemical fertilizers was the highest, its B coefficient was –0.002, and the significant level coefficient was 0.000, which was less than 0.05, indicating a significant effect. This shows that the increase in chemical fertilizer application is more conducive to the increase in food supply (Table 7).

In the analysis results of water yield service from 2000 to 2018, the order of influence on water yield service was as follows: implementation change in water conservation project > slope-to-ladder project implementation intention > change in implementation of restoring farmlands to forests projects > change in the implementation of slope-to-ladder projects > implementation intention of hydraulic engineering. Among them, the Wald value (46.218) of the implementation change in the water conservation project was the largest, the B coefficient was 0.088, and the significant level coefficient was 0.000, which is less than 0.05, indicating a significant impact. This indicates that the increase in the implementation of hydraulic engineering is more conducive to the reduction in water production, primarily due to increased evapotranspiration in the project areas such as water cellars and reservoirs, resulting in lesser water production (Table 7).

In the analysis results of soil conservation services from 2000 to 2018, the order of influence on soil conservation services was found to be as follows: change in human disturbance index > the change in the implementation of the restoring farmlands to forests project of > willingness in the implementation of the returning farmlands to forests projects > change in the implementation of the slope-to-ladder projects. Among them, the Wald value (41.562) of human disturbance index change was the largest, its B coefficient was 0.100, and the significant level coefficient was 0.000, which was less than 0.05, indicating a significant impact. This demonstrates that the increase in the human disturbance index will not be conducive to the increase in soil retention (Table 7).

In the analysis results of carbon sequestration services from 2000 to 2018, the order of the impact on carbon sequestration services was as follows: the change in human disturbance index > the change in the implementation of returning farmland to forestry projects > the willingness to implement the restoring farmlands to forests projects > change in agricultural population density. Among them, the Wald value (43.830) of the human disturbance index change was the largest, the B coefficient was 0.055, and the significant level coefficient was 0.000, which was less than 0.05, indicating a significant impact. This shows that the increase in the human disturbance index is not conducive to the increase in carbon sequestration (Table 8).

In the analysis results of habitat support service from 2000 to 2018, the order of the impact of habitat support services was as follows: change in human disturbance index > the willingness to implement the application of pesticides > the change in pesticide application amount > the implementation of the restoring farmlands to forests projects > the change in agricultural population density > the willingness to implement the restoring farmlands to forests projects. Among them, the Wald value (30.808) of the human disturbance index was the largest, its B coefficient was 0.018, and the significant level coefficient was 0.000, which was less than 0.05, indicating a significant impact. This shows that the increase in human disturbance is not conducive to the increase in habitat quality (Table 8).

In the analysis results of cultural services from 2000 to 2018, the order of influence on cultural services was as follows: change in the proportion of tertiary industry > aesthetic value > the change in the implementation the restoring farmlands to forests projects. Among them, the Wald value (18.471) of the proportion change in the tertiary industry was the largest, its B coefficient was –0.092, and the significant level coefficient was 0.000, which is less than 0.05, indicating a significant impact. This shows that the increase in the proportion of the tertiary industry is more conducive to the increase in the value of cultural services (Table 8).

## 4. Discussion

Selection of factors for ESS change drivers. Scholars mostly select land use change, policy and economic macro factors, such as population, policy and the level of economic development, while the micro-level driving factors are less selected, such as family ecological engineering compensation income, personal income, deforestation and so on. At present, the analysis of human driving factors affecting ESS change in karst mountainous areas is generally focused on macro factors, but there are few micro-level studies involving farmers’ behavior, attitude and willingness, which makes it difficult to reflect the impact of human driving factors on ESS change from the level of farmers’ behavior. Therefore, this study analyzes the impact of human activities on ESS from the perspective of farmers’ behavior, attitude and willingness through questionnaire data and research data, which strengthens the scientificity and comprehensiveness of the selection of human driving factors. The study uses multiple logistic regression model to analyze the driving factors affecting ESS change, not only from the macro factors such as population, economy and policy, but also from the macro factors such as population, economy and policy. The impact of human activities on ESS is also characterized by micro-factors such as farmers’ behavior, attitude and willingness. In terms of the research results of human driving factors of ESS change, many scholars have studied that the driving forces of ESS change are mainly macro factors such as ecological engineering, while in this study, besides the macro factors such as returning farmland to forestry, water conservancy, terracing and the proportion of tertiary industry are significant. The micro-factors such as the quantity and willingness of chemical fertilizer and pesticide, the quantity and willingness of terracing, the quantity of returning farmland to forest and aesthetic value are also significant.

With regard to the analysis method of driving mechanism of ESS change, at present, the main methods are principal component analysis and correlation analysis, while the multiple logistic regression model can divide the dependent variables and independent variables into continuous variables and classification variables, which is more suitable. Therefore, the multiple logistic regression model is used to quantitatively analyze the human driving factors of ESS change, and the most significant factors of ESS change in the study area are revealed. At the same time, the data of farmers’ policy implementation behavior, attitude and willingness are selected for quantitative analysis, which makes up for the lack of quantitative methods that cannot quantify farmers’ willingness, attitude and behavior, and makes the analysis results of ESS change impact mechanism more reasonable.

With regard to the ESS accounting method, InVEST and other models are used to quantitatively measure ESS, which have the advantages of less imported data and simplified processing of complex problems. The results of ESS accounting in this study are basically consistent with other related studies. Among them, the annual average food supply in the three periods keeps increasing year by year, which is consistent with the statistical yearbook of Guangnan County; the annual average soil conservation in the three periods is slightly different from the calculation results of Lang Yanqing in karst mountainous areas, which are also karst areas, and the overall results are different due to the spatial heterogeneity of the natural environment. The average water yield in the third period is slightly lower than that in the karst mountain area studied by Lang Yanqing. The reason is that the water yield is closely related to the precipitation, temperature and evapotranspiration in the area [55]. In this study, the spatial distribution of habitat quality value is higher in the area, and the study area is actually a large area of woodland with a good ecological environment, so the results are more consistent with the actual situation. In a word, due to different study areas, the results of the study will be different, but the results of ESS accounting in the southwest of the same latitude zone are not very different. Therefore, it is considered that the ESS accounting results are feasible.

## 5. Conclusions

InVEST and other models were used to evaluate six ESS in three periods, analyze the spatial and temporal variation characteristics of each ESS, explore the impact of human activity interference on each ESS, and identify the most significant driving factors of each ESS change. The main conclusions are as follows:

Temporally, the annual average food supply and cultural value increased year by year, while the annual average habitat quality decreased year by year; the annual average water production, soil conservation and carbon sequestration decreased first and then increased year by year; spatially, the food supply, cultural value and water production increased more in the middle, while the soil conservation showed a roughly opposite trend. Carbon sequestration and habitat quality increased more in the east.

The implementation of major ecological projects such as reforestation, slope-to-ladder conversion, biogas and water conservancy is beneficial to some extent to increase food supply, soil conservation, water production, carbon sequestration, habitat support and cultural services. Among them, the change in water conservation project implementation has the most significant impact on water production services; the decrease in agricultural population share is beneficial to habitat support services; and the increase in annual per capita tourism income and the increase in tertiary industry share are beneficial to the increase in cultural services. Among them, the change in the proportion of tertiary industry affects cultural services most significantly. Changes in anthropogenic disturbance index affect soil conservation, carbon sequestration and habitat support services most significantly, respectively.

The increasing pesticide and fertilizer application and the increasing willingness of and number of new farmers using pesticides and fertilizers positively affect food supply services, but are detrimental to habitat quality. Among them, willingness to implement pesticides affects habitat support services most significantly, and willingness to implement chemical fertilizers affects food supply services most significantly. The level of farmers’ participation in reforestation and water conservation projects and their willingness to participate in them are increasing, which positively affect soil conservation, water production and carbon sequestration services. Among them, the willingness to implement reforestation projects has the most significant impact on carbon sequestration services. Farmers’ participation in slope-to-ladder and biogas projects is, to a certain extent, beneficial to the increase in soil conservation and carbon sequestration services. Among them, the willingness to implement the slope-to-ladder project affects water production services and soil conservation most significantly, and the aesthetic value affects cultural services most significantly.

## Figures and Tables

**Table 1:** Data source and processing.

Data Name	Data Format	Resolution	Data Source
Land cover data	tif	30 m	https://zenodo.org/record/5210928 (accessed on 30 March 2020)
Digital terrain data	tif	30 m	http://www.gscloud.cn/ (accessed on 4 April 2020)
Meteorological data	nc	1 km	http://www.geodata.cn/ (accessed on 6 April 2020)
Soil data	mdb	1 km	https://www.fao.org/ (accessed on 6 April 2020)
Population count	tif	100 m	https://www.worldpop.org/ (accessed on 8 April 2020)
GDP	tif	1 km	http://www.resdc.cn/ (accessed on 10 April 2020)
NDVI data	tif	30 m	http://www.gscloud.cn/ (accessed on 4 April 2020)
Tourism Data of Guangnan County	word		Statistics Bureau and Tourism Bureau of Guangnan County

**Table 2:** Time variation characteristics of each ESS.

	2000	2010	2018	2000–2010	2010–2018	Average Annual Variation
food supply (t/ha)	1.79	2.62	3.38	0.83	0.76	1.59
water yield (mm)	763.68	685.94	780.71	−77.74	94.77	17.03
soil conservation (t/ha)	821.92	764.31	951.59	−57.61	187.28	129.67
carbon sequestration (CNY·ha^−^^1^·a^−^^1^)	1578.71	1398.62	1494.38	−180.09	95.76	−84.33
habitat quality	0.77	0.72	0.70	−0.05	−0.02	−0.07
cultural value (CNY/ha)	4.88	5.84	9.49	0.94	3.65	4.61

**Table 3:** Changes in the number of participating farmers after the implementation of the project.

	Number of Farm Households	Slope-to-Ladder Project	%	Fallow Forestry Project	%	Hydraulic Engineering	%	Biogas Project	%
Project Implementation									
After implementation		72	17.60	70	17.19	125	30.71	79	16.89
Status Quo		63	15.40	210	51.59	362	88.94	18	3.84
Variance		−9	2.20	140	34.40	237	58.23	−61	13.03

**Table 4:** Number of households using pesticides and fertilizers and change in application Amount.

Year	Number of Households Using Pesticides	Proportion	Number of Households Using Chemical Fertilizer	Proportion	Pesticide Application Amount of Cultivated Land (ml/mu)	Fertilizer Application Amount of Cultivated Land (kg/mu)
2000	166	40.78	178	37.80	75	15
2010	221	54.29	246	60.44	350	35
2018	392	96.31	401	98.52	900	70

**Table 5:** Selection and Interpretation of Independent Variable Driving Factors.

Driving Factors	Factor Category	Macro Drivers (2000–2018)	Micro Drivers (2000–2018)
Social factors	Population	X1: population density change	
		X2: changes in agricultural population density	
	Ecological Engineering	X3: implementation change in slope-to-ladder project	
		X4: changes in the implementation of water conservancy projects	
		X5: change in returning farmland to forest project	
		X6: biogas engineering change	
Behavioral factors	Behavior and farmers’ willingness	X7: change in human disturbance index	X8: change in pesticide application
			X9: change in fertilizer application
			X10: willingness to implement chemical fertilizer X11: Pesticide implementation intention
			X12: willingness to implement biogas project
			X13: implementation intention of changing slope to ladder
			X14: willingness to implement water conservancy projects
			X15: implementation intention of returning farmland to forest project
Economic factors	Economic development	X16: changes in farmers’ net income	-
		X17: changes in the proportion of primary industry	
		X18: changes in the proportion of secondary industry	
		X19: changes in the proportion of tertiary industry	
Cultural factor	Cultural tourism	-	X20: tourism value
			X21: aesthetic value
			X22: educational value
			X23: historical and cultural heritage value

Note: “-” indicates that the relevant driving factor is not selected.

**Table 6:** Fitting information of various service models from 2000 to 2018.

Model	Model Fitting Standard	Likelihood Ratio Test		
	Twice Log Likelihood	Chi Square	df	Significant Level
Food supply	22,280.070	194.913	36	0.000
Water yield	50,518.139	548.426	34	0.000
Soil conservation	51,030.816	583.451	34	0.000
Carbon sequestration	57,317.560	354.125	34	0.000
Habitat quality	53,493.966	512.510	34	0.000
Cultural value	39,923.621	320.640	42	0.000

**Table 7:** Model results for food availability and water production and soil retention, 2000–2018.

Independent Variable	2000–2018 Decrease in Food Supply			2000–2018 Decreased in Water Production Decreased			2000–2018 Decrease in Soil Conservation		
	B	Wald	Significant Level	B	Wald	Significant Level	B	Wald	Significant Level
Intercept	−0.597	6.688	0.010	−2.621	13.692	0.001	−2.640	29.698	0.000
X2	−0.118	0.191	0.081	−0.008	0.057	0.755	−0.056	1.316	0.135
X3	−3.515	3.648	0.001	0.009	10.353	0.003	−0.308	6.015	0.003
X4	−0.535	1.892	0.169	0.088	46.218	0.000	−0.118	2.649	0.063
X5	0.062	0.002	0.966	0.260	11.097	0.002	−0.030	30.001	0.000
X6	−0.308	0.681	0.409	−0.189	0.220	0.655	0.970	0.838	0.360
X7	0.148	0.554	0.110	0.017	0.108	0.742	0.100	41.562	0.000
X8	−0.002	21.051	0.000	−0.004	0.983	0.320	0.011	1.427	0.128
X9	−0.002	21.051	0.000	−0.004	0.983	0.320	0.027	0.564	0.573
X10	−0.160	0.384	0.005	−0.197	0.705	0.413	−0.249	1.642	0.126
X11	−0.070	6.000	0.000	0.403	1.850	0.178	−0.560	2.546	0.073
X12	0.003	0.246	0.620	−0.101	0.142	0.706	−0.098	2.101	0.088
X13	0.080	0.578	0.447	−0.020	36.921	0.000	0.371	18.992	0.001
X14	−0.090	0.203	0.138	−0.017	8.142	0.006	−0.399	1.938	0.091
X15	0.136	0.557	0.092	−0.126	0.030	0.791	0.667	13.538	0.002
X16	−0.070	0.534	0.465	0.000	0.323	0.570	0.002	0.334	0.678
X17	−0.011	5.155	0.004	0.295	0.516	0.473	−0.090	1.203	0.138
X18	−2.835	0.751	0.386	0.196	0.139	0.709	0.182	2.155	0.076
X19	0.200	0.367	0.544	0.477	2.557	0.110	−0.626	0.687	0.420

**Table 8:** Model results for carbon sequestration and habitat quality and cultural services, 2000–2018.

Independent Variable	2000–2018 Decrease in Carbon Sequestration			2000–2018 Decrease in Habitat Quality			2000–2018 Decrease in Cultural Services		
	B	Wald	Significant Level	B	Wald	Significant Level	B	Wald	Significant Level
Intercept	−0.187	1.023	0.312	−0.844	2.539	0.111	2.542	12.211	0.000
X2	0.016	4.384	0.007	−0.001	0.072	0.789	−0.359	0.532	0.071
X3	0.002	2.758	0.076	−0.086	0.106	0.745	0.136	1.557	0.241
X4	0.015	1.310	0.252	−0.164	1.569	0.210	−0.365	0.280	0.401
X5	−2.256	12.121	0.000	−3.350	4.555	0.018	−3.013	2.526	0.007
X6	0.538	1.793	0.181	−0.119	0.211	0.137	0.136	1.557	0.384
X7	0.055	13.830	0.000	0.018	10.808	0.000	0.560	1.434	0.102
X8	0.001	1.113	0.095	0.002	5.635	0.004	−0.092	1.471	0.062
X9	0.001	1.319	0.131	−0.003	2.288	0.012	−0.007	1.906	0.420
X10	−0.226	0.285	0.213	−0.220	1.258	0.071	−0.207	0.452	0.374
X11	0.041	0.212	0.645	0.094	6.119	0.003	−0.411	0.550	0.187
X12	−0.003	0.243	0.622	0.048	0.376	0.540	0.454	1.684	0.055
X13	0.423	1.951	0.124	−0.028	0.055	0.815	0.150	0.724	0.099
X14	0.088	1.939	0.101	−0.328	1.585	0.215	2.118	0.820	0.365
X15	0.523	13.032	0.000	0.611	5.867	0.015	−0.055	0.213	0.644
X16	0.000	0.036	0.850	0.001	0.911	0.310	0.027	0.006	0.098
X17	0.179	0.584	0.445	−0.066	0.320	0.572	1.238	0.005	0.341
X18	−0.015	1.500	0.471	0.124	2.560	0.110	0.115	0.101	0.750
X19	−0.001	1.341	0.261	0.414	0.891	0.149	−0.092	8.471	0.000
X20							0.136	6.906	0.000
X21							−0.272	1.608	0.138
X22							−0.032	0.393	0.531
X23							−0.253	0.955	0.475

## Data Availability

All data generated and analyzed during this study are included in this article.
